# Memory-Guided Saccades in Psychosis: Effects of Medication and Stimulus Location

**DOI:** 10.3390/brainsci11081071

**Published:** 2021-08-16

**Authors:** Eleanor S. Smith, Trevor J. Crawford

**Affiliations:** 1 Department of Psychology, University of Cambridge, Cambridge CB2 3EB, UK; 2 Department of Psychology, Centre for Ageing Research, Lancaster University, Lancaster LA1 4YF, UK; t.crawford@lancaster.ac.uk

**Keywords:** memory-guided saccades, schizophrenia, bipolar disorder, positive symptoms, negative symptoms

## Abstract

The memory-guided saccade task requires the remembrance of a peripheral target location, whilst inhibiting the urge to make a saccade ahead of an auditory cue. The literature has explored the endophenotypic deficits associated with differences in target laterality, but less is known about target amplitude. The data presented came from Crawford et al. (1995), employing a memory-guided saccade task among neuroleptically medicated and non-medicated patients with schizophrenia (*n* = 31, *n* = 12), neuroleptically medicated and non-medicated bipolar affective disorder (*n* = 12, *n* = 17), and neurotypical controls (*n* = 30). The current analyses explore the relationships between memory-guided saccades toward targets with different eccentricities (7.5° and 15°), the discernible behaviour exhibited amongst diagnostic groups, and cohorts distinguished based on psychotic symptomatology. Saccade gain control and final eye position were reduced among medicated-schizophrenia patients. These metrics were reduced further among targets with greater amplitudes (15°), indicating greater deficit. The medicated cohort exhibited reduced gain control and final eye positions in both amplitudes compared to the non-medicated cohort, with deficits markedly observed for the furthest targets. No group differences in symptomatology (positive and negative) were reported, however, a greater deficit was observed toward the larger amplitude. This suggests that within the memory-guided saccade paradigm, diagnostic classification is more prominent in characterising disparities in saccade performance than symptomatology.

## 1. Introduction

Research published over the past decade has highlighted and established an encouraging novel area of research which considers the oculomotor system as an effector system for visual memory ([1], for review). Research exploring the expression of memory through eye movements demonstrate how they are not simply a passive reflection of memory, but rather play an active role in contributing to the formation and retrieval of information [2].

The memory-guided saccade task is a highly informative paradigm, which requires remembering the location of a peripheral target, whilst inhibiting the urge to make a saccade to that target ahead of an auditory cue. Typically, individuals would produce a reflexive eye movement in response to a novel stimulus, but in the memory-guided saccade paradigm they are asked to suppress and delay their saccade until the presentation of a cue. Whilst suppressing this saccade, fixation should be maintained at the centre of the display while simultaneously encoding the spatial location of a peripheral target stimulus. During the delay period before receiving the cue, cells in the superior colliculus respond to the continued presence of the fixation stimulus [3,4], facilitating inhibition and maintaining the spatial representation of the peripheral location. Upon receiving the cue, a volitional saccade to the remembered target ensues. No visual information is provided on the location of the previously presented peripheral target at the moment of saccade initiation. This paradigm, therefore, examines the inhibition of a reflexive action, the ability to generate an internal representation of space (spatial working memory), and the inhibition of the saccadic motor program during the memorisation process. A major advantage of the oculomotor system is that detailed neurophysiological and biochemical operations can be explored using precise neuronal activity in equivalent or identical paradigms in animal studies. In seminal work, Goldman-Rakic and colleagues [5] demonstrated that D1 neurons in the dorsolateral pre-frontal cortex (DLPFC) played a critical role in memory-guided oculomotor controls, and the generation of representations in working memory. Research revealed that following inactivation of DLPFC with muscimol (a selective agonist for the GABA_A_ receptors), memory-guided saccades become hypometric and inaccurate in a spatially specific manner [6]. While the DLPFC [5] and substantia nigra pars reticulata (SNpr) [7] are implicated in the short-term memory buffer utilised during the memory-guided saccade task, the parahippocampal cortex (PHC) may also be implicated in a varied version of the memory-guided saccade task. Research has shown that accuracy of memory-guided saccades, when memorisation delays are extended to between one and twenty seconds, depends on the DLPFC [8], whilst the PHC is thought to be responsible for accuracy when the delays are longer than twenty-seconds (and up to a few minutes) [8,9,10,11].

Saccadic eye movements are a ubiquitous form of information gathering (demonstrated in primates) [12,13] and play a role in modulating ongoing neural activity in the primate hippocampus [14,15,16]. However, Ryan et al. [1] highlight that there are no known direct connections between hippocampal subfields and the oculomotor system. Thus, they illustrated by examining whole-cortex connectivity in a model-based approach that there is an extensive set of polysynaptic pathways mediating the exchange of information between the oculomotor and memory systems [17]. Considering the functional dynamics and recurrent interactions of the network involved in the hippocampal guidance of ocular control, they highlight that neural activity in these areas, are important for the cognitive and motoric control of eye movements [18]. Moreover, electrophysiological studies in humans and primates [19,20], highlight that hippocampal memory representations are used to guide saccades to behaviourally relevant locations [21]. With relevance to the present research, visual exploration of novel, but not repeated stimuli result in a reset of hippocampal theta oscillations [20], thus the consistency of this ability predicts the success of novel memory encoding [22]. Given the role of the prefrontal cortex and schizophrenia pathology and treatment, the memory-guided saccade task employed, with small memorisation periods and repeated stimulus presentation, would be expected to detect neurocognitive impairment in patients with schizophrenia.

Previous research has explored the memory-guided saccade paradigm in relation to the psychosis-continuum, highlighting a series of deficits distinguishing patients from neurotypical controls ([23,24], for example). In memory-guided saccades, schizophrenia patients have been shown to elicit increased latencies and/or reduced gain control and final eye positions [24,25,26,27,28,29,30,31] as well as elevated disinhibition errors [23]. Decreased gain control has been reported among psychosis patients and their relatives [25,26,30], as well as increased latencies [24,27] among people with schizophrenia compared to controls. These deficits have been shown to predate the onset of the disease [32,33]. This atypicality has been replicated in psychosis patients [23,24,27,28,34] independent of medication status [28], their full siblings [26], relatives [27,35], and those with high-schizotypy [36,37,38]; supporting its candidacy as an oculomotor endophenotype.

All research cited thus far has largely overlooked the potential role of spatial location. Given that targets closer to the fovea are more likely to capture attention due to the cortical magnification factor and increased salience. If near targets require greater levels of inhibition to avoid attentional capture, target eccentricity (how far away the remembered target is from central fixation) may be expected to be a highly relevant factor. This may also interact with symptomatology given that prefrontal impairment has been reliably associated in schizophrenia (SZ) patients with predominantly negative symptoms [39,40,41]. There is evidence to suggest that differences may be observed when exploring these deficits in accordance with target eccentricity [42,43]. Existing research has demonstrated deficits among patients with a family history of schizophrenia at smaller angles than patients without a family history of this disease [42]. It is therefore relevant to explore these deficits at different target eccentricities as this may provide higher specificity in detecting a marker of vulnerability in schizophrenia [26]. The current work explores the dataset reported by [23], to examine the effect target eccentricity (near vs. far targets) has on performance in the memory-guided saccade task.

There are very few studies investigating the influence of target eccentricity in the memory-guided saccade task, especially within psychosis patients. A key study, however, is reported by Landgraf et al. [26] who explored memory-guided saccade metrics among schizophrenia patients (*n* = 16), their family members (*n* = 19) and a control group (*n* = 18). Data reported a reduction in errors from 46.2% at ‘small stimulus eccentricities’ (4°, 6°, 8°) to 5.6% at ‘large stimulus eccentricities’ (10°, 12°) among controls. This finding suggests that controls are more susceptible to distractors that are closer to a central fixation point, however, among the schizophrenia patients and their family members, no difference in error production was reported. In alignment with the present research, Landgraf et al. [26] explored primary saccade gain control and final eye position metrics, where schizophrenia patients exhibited reduced performance when compared to their control counterparts but overlooked the potential mediating effect of target eccentricity. A conclusion of this study was that the memory-guided saccade paradigm provided a more specific way of identifying saccadic abnormalities than other saccade paradigms, with involuntary errors more prevalent in schizophrenia patients and their full-siblings, even at the largest target amplitudes. They concluded that the paradigm could be utilised in identifying saccadic abnormalities in psychoses. The present research therefore aims to expand on this analysis; investigating whether primary saccade gain control and final eye position metrics are affected by target eccentricity.

The present research also incorporates both schizophrenia and bipolar disorder (BD) patients, to explore their comparative oculomotor responses to the memory-guided saccade task. There is very little research exploring memory-guided saccades among bipolar disorder patients. Crawford et al. [23,44] illustrated no group differences (between schizophrenia, bipolar disorder, neurotics, and controls) in the latency metric, but trends towards significant reductions in gain control and final eye position among schizophrenia patients. In a systematic review, Carvalho et al. [45] did not report any literature exploring memory-guided saccades among individuals with bipolar disorder. Moreover, the schizophrenia cohort reported in the study carried out by Landgraf et al. [26] were all medicated; thus, the present manuscript will therefore explore memory-guided saccades among medicated and non-medicated individuals diagnosed with bipolar affective disorders, comparing their oculomotor behaviours with those exhibited by medicated and non-medicated individuals with schizophrenia.

A distinct limitation of the Landgraf et al. [26] paper is the lack of reference to symptomatology. The symptomatology in the present research reflects on positive symptoms; which describe an excess or distortion of normal mental function (for example, delusions, hallucinations, disorganized behaviour), and negative symptoms, which refer to a diminution or absence of normal mental or psychological functions related to motivation and interest (for example, anhedonia, asociality, and psychomotor poverty) or expression (for example, blunted affect [46]). Existing research has highlighted significant associations between patients with negative symptoms (as measured by either the SANS scale [47] or the PANSS [48]) and greater suppression errors in the antisaccade task [28,49,50,51]. In a small study (*N* = 21) Winograd-Gurvich et al. [52] explored the influence of negative symptoms amongst a schizophrenia patient sample on a series of saccade tasks; one of which included a memory-guided task. They describe how the high-negative symptom group made a significantly greater percentage of errors than controls, with the memory-guided saccade paradigm producing significantly longer latencies than other saccade tasks, but there were no clear differences between the high and low negative were reported. No group differences were reported for gain control or final eye position measures; two metrics thought to be important in psychotic saccade deficits [53]. However, this study appears to be underpowered with only 10 and 11 participants per group. More recent research has highlighted the existence of different relationships between the symptoms of schizophrenia and saccade task performance. Obyedkov et al. [54] in a much larger study (*N* = 156) highlighted that among schizophrenia patients and those at ultra-high risk for psychosis, the negative symptoms but not positive or disorganized symptoms were associated with atypical latencies in predictive and reflexive saccade tasks. This was replicated by Smith and Crawford [53] who found significant hypometria and lower gain control amongst those patients experiencing predominantly high-negative symptoms in a predictive saccade task. Based on the existing evidence showing greater deficit among those with predominantly negative traits, the current work explores whether this is true for the deficits observed in the memory-guided saccade task. To our knowledge this is the first study of the influence of symptomatology on the production of memory-guided saccades amongst schizophrenia and bipolar disorder patients.

In this work, we will address the extent to which target eccentricity influences latency, gain control, and final eye position measures in the memory-guided saccade task amongst schizophrenia and bipolar affective patients, as well as control participants. We also aim to address the influence medication may have on performance, and whether symptomatology can distinguish between typical saccadic behaviour and deficit. Answering these questions will further our understanding of essential features for the development of oculomotor biomarkers, which can be utilised in the early diagnosis of psychosis.

## 2. Materials and Methods

### 2.1. Participant Selection

The dataset presented in this research came from the Crawford et al. [23,44] studies. The participant groups consisted of medicated schizophrenia patients (M-SZ; *n* = 40; 21 male and 19 female; mean age ± SD = 40 ± 12 years, range = 22–61 years), non-medicated schizophrenia patients (NM-SZ; *n* = 18; 17 male and 1 female; mean age ± SD = 39 ± 13 years, range = 20–61 years), medicated bipolar affective disorder patients (M-BD; *n* = 14; 7 male and 7 female; mean age ± SD = 44 ± 12 years, range = 20–60 years), non-medicated bipolar affective disorder patients (NM-BD; *n* = 18; 12 male and 6 female; mean age ± SD = 42 + 12 years, range = 20–60 years), and controls (CON; *n* = 31; 16 male and 15 female; mean age ± SD = 39 ± 11 years, range = 25–57 years). All patients were identified from the case notes of out-patients at the Royal London Hospital and DSM-III-R criteria [55]. Controls were recruited from among all grades of hospital staff. Informed consent was obtained from all the participants and the study was approved by the Tower Hamlets Ethical Committee. The CONs and their immediate families lacked any history of mental disease. Group matching, exclusion criteria, and further details of the recruitment procedures and clinical assessments are reported in Supplementary Materials (S1) and Crawford et al. [23,44]. Group means and standard deviations on all clinical measures collected in Table 1. A one-way ANOVA showed no difference in age between the diagnostic and control groups (F(4,118) = 0.546, *p* = 0.70).

To address the symptomatology of the participant groups, The Scale for the Assessment of Negative Symptoms and the Scale for the Assessment of Positive Symptoms (SANS and SAPS) were employed. SANS measures negative symptoms on a 25 item, 6-point scale [47]. Items are listed under five domains of affective blunting, alogia, avolition/apathy, anhedonia/asociality, and attention. SAPS measures positive symptoms on a 34 item, 6-point scale [48]. Items are listed under hallucinations, delusions, bizarre behavior, and positive formal thought disorder.

### 2.2. Measurement of Saccades

Apparatus set-up, procedure and the predictive saccade paradigm are reported in the Supplementary Materials (S1) and reported in Crawford et al. [23,44].

**Figure 1:**
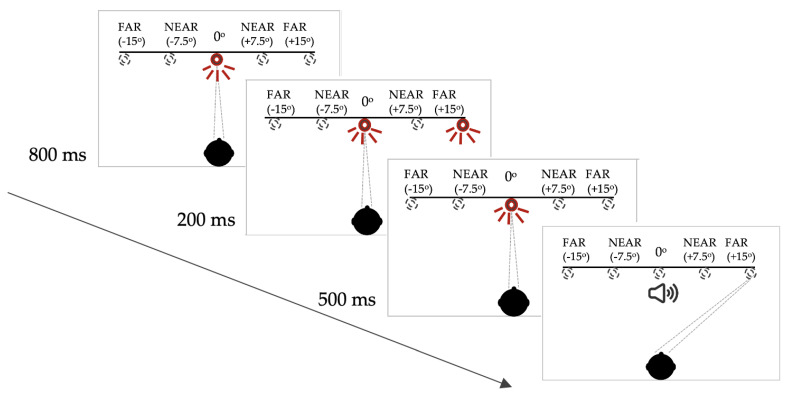
Saccadic target paradigm for the memory-guided saccade task. Note: the targets were only visible when lit up. See Supplementary Materials (S1) for full description of the target configuration and the subject instructions.

For this memory-guided saccade task, each trial began with an illuminated central LED. After 800 milliseconds (ms), a peripheral LED flashed on for 200 ms. The central LED remained on, however, and the participant was instructed not to look towards the peripheral LED immediately. The central LED was extinguished 500 ms after offset of the peripheral target and, at this point, the participant was required to make a saccade to the remembered location of the previously illuminated peripheral LED. In this paradigm, the buzzer onset that provided the temporal cue, was coincident with the offset of the central (fixation) LED (see Figure 1 for schematic representation of this paradigm).

### 2.3. Data Processing and Statistical Analysis

In the memory-guided saccade paradigm, the latency and spatial accuracy or gain (i.e., saccade eccentricity/target eccentricity) of the initial saccade and the gain of the final eye position (FEP) on each trial was analysed. The mean and standard deviation of each parameter was calculated for the four target locations. In the current paradigms, the number of trials on which a failure of saccadic suppression occurred was tallied for each participant. In calculating latency and gain, these erroneous responses were discarded. In assessing saccadic distractibility, an error was registered if a saccade occurred during the 500ms delay between target offset and the imperative cue, buzzer onset (accompanied by fixation LED offset). For the target eccentricity analyses, the ‘Near’ targets were classified as those at ±7.5° and the ‘Far’ targets were classified as those at ±15°. ±7.5° and ±15° were chosen to address previous studies which have used smaller target amplitude (e.g., 2°) separations which are ambiguous in whether they are classified as ‘near’ or ‘far’.

## 3. Results

### 3.1. Diagnosis-Based Analysis

For the diagnostic-based analyses, participants were separated into M-SZ *(n* = 29), NM-SZ (*n* = 11), M-BD (*n* = 12), NM-BD (*n* = 17), and CON (*n* = 30). To explore the effect of neuroleptic medication on performance in the memory-guided saccade task, diagnostic groups (SZ and BD) were collapsed into whether they were neuroleptic medicated (*n* = 41) or not on neuroleptic medication (*n* = 68).

#### 3.1.1. Saccade Primary Saccade Gain

**Figure 2:**
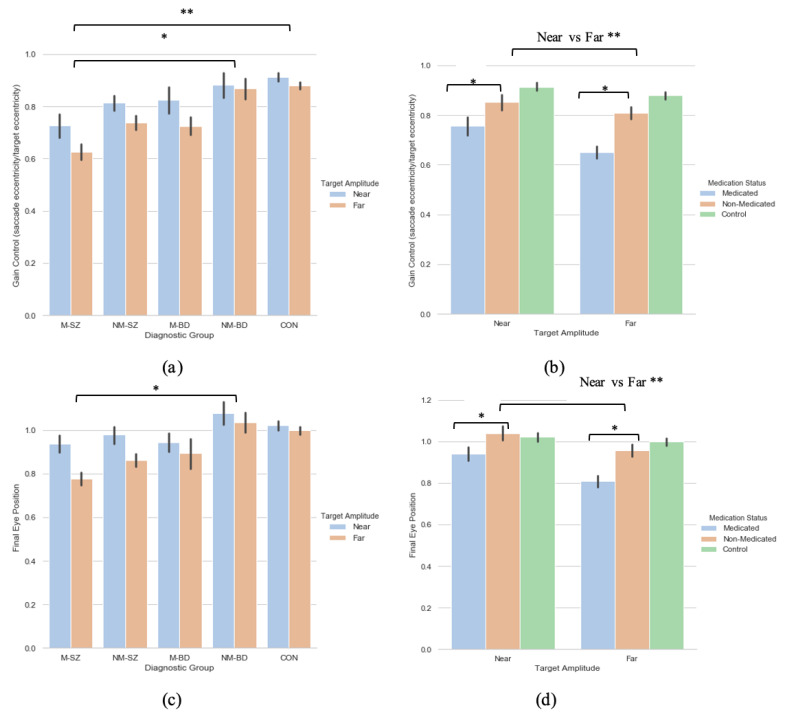
A comparison of performance in the (**a**) gain and (**c**) final eye position measures between the two target eccentricities within each diagnostic group; a comparison of performance in the (**b**) gain and (**d**) final eye position measures between the two peripheries between the medicated and non-medicated cohorts (collapsed across diagnostic category). For reference, the control cohort are included here but were not included in the reported analysis. * Refers to significance at the *p* < 0.05 level. ** Refers to significance at the *p* < 0.01 level. Error bars refer to standard error.

A 2 (target eccentricity: Far, Near) × 5 (Group: M-SZ, NM-SZ, M-BD, NM-BD, CON) repeated-measures ANOVA was carried out on the primary saccade gain data with Bonferroni post hoc tests for multiple comparisons, where main effects of target eccentricity (F(1,93) = 8.905, *p* = 0.004, η_p_^2^ = 0 087) and group (F(4,93) = 4.920, *p* < 0.001, η_p_^2^ = 0.175) were found (see Figure 2a). Bonferroni post hoc analyses for multiple comparisons illustrated how these distinctions were driven by differences between the M-SZ vs. CON (*p* < 0.001) and M-SZ vs. NM-BD (*p* = 0.019) groups (see Figure 2a). Gain control in the M-SZ group was therefore significantly reduced when contrasted with the NM-BD and CON groups. To explore the effect of neuroleptic medication, a two (target eccentricity: Far, Near) by two (medication Status: M, NM) repeated-measures ANOVA was executed, where a main effect of target eccentricity (F(1,106) = 13.979, *p* < 0.001, η_p_^2^ = 0.117) and medication status (F(1,106) = 16.181, *p* < 0.001, η_p_^2^ = 0.132) was found. This relationship suggests that the medicated cohort produced significantly lower gain data in both eccentricities compared to the non-medicated cohort (see Figure 2b).

#### 3.1.2. Saccade Final Eye Position

A two (target eccentricity: Far, Near) by five (group: M-SZ, NM-SZ, M-BD, NM-BD, CON) repeated-measures ANOVA was performed on the FEP data, where a main effect of target eccentricity (F(1,93) = 9.253, *p* = 0.003, η_p_^2^ = 0.090) and group (F(4,93) = 3.065, *p* = 0.020, η_p_^2^ = 0.116) were found. Bonferroni post hoc analyses for multiple comparisons illustrated how these distinctions were driven by differences between the M-SZ and NM-BD (*p* = 0.033) groups (see Figure 2c. FEP in the M-SZ group was, therefore, significantly reduced when contrasted with the NM-BD group. To explore the effect of neuroleptic medication, a two (target eccentricity: Far, Near) by two (medication status: M, NM) repeated-measures ANOVA was carried out. A main effect of target eccentricity (F(1,106) = 16.345, *p* < 0.001, η_p_^2^ = 0.134) and medication status (F(1,106) = 10.677, *p*< 0.001, η_p_^2^ = 0.092) was observed. This relationship suggests that the medicated cohort displayed greater hypometria in both eccentricities compared to the non-medicated cohort (see Figure 2d). A target eccentricity*medication status interaction was also found (F(1,106) = 3.858, *p* = 0.05, η_p_^2^ = 0.035).

#### 3.1.3. Saccade Latency

A two (target eccentricity: Far, Near) by five (group: M-SZ, NM-SZ, M-BD, NM-BD, CON) repeated-measures ANOVA was carried out, where no significant main effects were observed. To explore the effect of neuroleptic medication, a two (target eccentricity: Far, Near) by two (medication status: M, NM) repeated-measures ANOVA was executed on the data. No significant main effects or interactions were observed. This is consistent with previous research [10,26] revealing that saccade parameter of primary saccade latency is relatively insensitive to psychosis, neuroleptic or target eccentricity.

In sum, no effects of target eccentricity were observed for the latency metric. This is analogous with the existing literature [10,38]. Landgraf and colleagues [26] highlighted an overall group difference in gain control and final eye position; whereby the SZ patients showed reduced abilities when compared to their family members and controls. The present research mimics this notion but develops the idea further. Consequently, gain control and final eye position in the M-SZ patients was significantly diminished in contrast to the NM-BD and CON groups. Moreover, these measures were lessened further among targets with greater eccentricities (15°). This finding was paralleled again when investigating the effect of neuroleptic medication: expectedly, the medicated cohort (regardless of diagnosis) exhibited reduced gain control and final eye positions in both eccentricities compared to the non-medicated cohort, but this deficit is markedly observed in the furthest targets.

### 3.2. Symptoms-Based Analysis (1)

To establish that collapsing near and far targets from both the left and right literalities would not add any uncertainties to the data, a two (laterality: left, right) by two (group: high-SANS, low-SANS) repeated measures ANOVA was explored; illustrating no significant effects for latency, gain or FEP in either laterality (*p* > 0.5). Thus, the analyses include ‘Near’ targets (those at ±7.5°) and ‘Far’ targets (±15°).

For the symptom-based analyses, a median split was used to divide the participants into a high- (score > 18) and low- (score < 18) negative/positive symptoms group. Initially, groups with high-/low- negative symptoms or high-/low- positive symptoms were collapsed across diagnosis to explore the singular influence of positive and negative symptomatology (regardless of diagnostic classification) on memory-guided saccade performance. The constitution of the cohort was as follows: high-SANS (*n* = 24), low-SANS (*n* = 39), high-SAPS (*n* = 37) and low-SAPS (*n* = 27).

#### 3.2.1. Primary Saccade Gain

**Figure 3:**
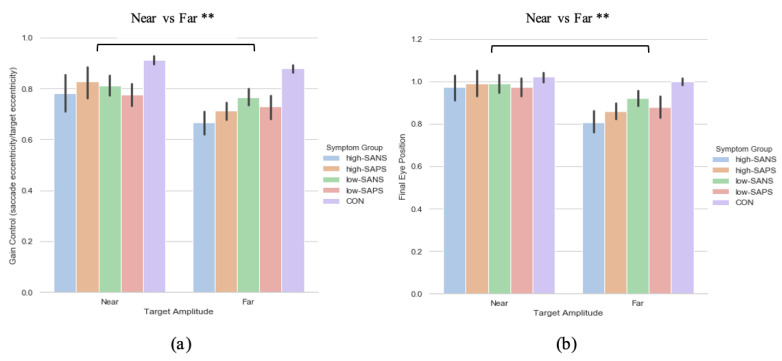
A comparison of performance in the (**a**) gain and (**b**) final eye position measures between the two target eccentricities based on their negative and positive symptoms. ** Refers to significance at the *p* < 0.01 level. Error bars refer to standard error.

A two (target eccentricity: Far, Near) by two (group: high-SANS, low-SANS) repeated measures ANOVA was carried out. This analysis highlighted a main effect of target eccentricity (F(1,61) = 7.766, *p* = 0.007, η_p_^2^ = 0.113; Figure 3a). When looking at the descriptive statistics, it appears that differences between high- and low-SANS groups are more apparent for the furthest target, but no significant group effect was found. To explore this potential relationship, a multiple regression was performed to predict group membership (high vs. low negative groups, collapsed across diagnostic label) from gain control towards the furthest target and gain control towards the nearest target. These variables did not significantly predict group membership (F(2,60) = 1.41, *p* = 0.252, *R*^2^ = 0.045). A two (target eccentricity: Far, Near) by two (group: high-SAPS, low-SAPS) repeated measures ANOVA was also carried out; displaying a main effect of target eccentricity also (F(1,62) = 7.412, *p* = 0.008, η_p_^2^ = 0.107; Figure 3b). For both analyses, gain control displayed greater reductions towards the furthest target eccentricity. No group differences were observed for either analysis. A multiple regression was performed to predict group membership (high vs. low positive groups, collapsed across diagnostic label) from gain control towards the furthest target and gain control towards the nearest target. These variables did not significantly predict group membership (F(2,61) = 0.274, *p* = 0.76, *R*^2^ = 0.009).

#### 3.2.2. Saccade Final Eye Position

A two (target eccentricity: Far, Near) by two (group: high-SANS, low-SANS) repeated measures ANOVA was carried out. The data reports a main effect of target eccentricity in FEP (F(1,61) = 11.192, *p* < 0.001, η_p_^2^ = 0.155; Figure 3b), whereby more variability is observed in the furthest target. To explore this idea, a multiple regression was performed to predict group membership (high vs. low negative groups, collapsed across diagnostic label) from FEP towards the furthest target and FEP towards the nearest target. These variables did not significantly predict group membership (F(2,60) = 1.66, *p* = 0.199, *R*^2^ = 0.052). A two (target eccentricity: Far, Near) by two (group: high-SAPS, low-SAPS) repeated measures ANOVA was also executed for the high-/low-SAPS groups; showing a main effect of target eccentricity in FEP (F(1,62) = 10.048, *p* = 0.002, η_p_^2^ = 0.139; Figure 3b). For both analyses, FEP showed larger reductions towards the furthest most target. No group differences were observed for either analysis. A multiple regression was performed to predict group membership (high vs. low positive groups, collapsed across diagnostic label) from FEP towards the furthest target and FEP towards the nearest target. These variables did not significantly predict group membership (F(2,61) = 0.103, *p* = 0.902, *R*^2^ = 0.003).

#### 3.2.3. Saccade Latency

A two (target eccentricity: Far, Near) by two (group: high-SANS, low-SANS) repeated measures ANOVA was performed; showing no significant effect of the latency metric. The same analysis (a two (target eccentricity: Far, Near) by two (group: high-SAPS, low-SAPS) repeated measures ANOVA) was carried out for the high- and low-SAPS groups, displaying the same result.

### 3.3. Symptoms-Based Analysis (2)

To investigate the effect of symptomatology between diagnostic categories, a similar analysis was repeated. Here, participants were grouped based on their high-/low- positive/negative symptom scores and were then divided by diagnostic category: high-SANS (SZ *n* = 13; BD *n* = 11), low-SANS (SZ *n* = 11; BD *n* = 28), high-SAPS (SZ *n* = 20; BD *n* = 17), low-SAPS (SZ *n* = 11; BD *n* = 16).

#### 3.3.1. Primary Saccade Gain

**Figure 4:**
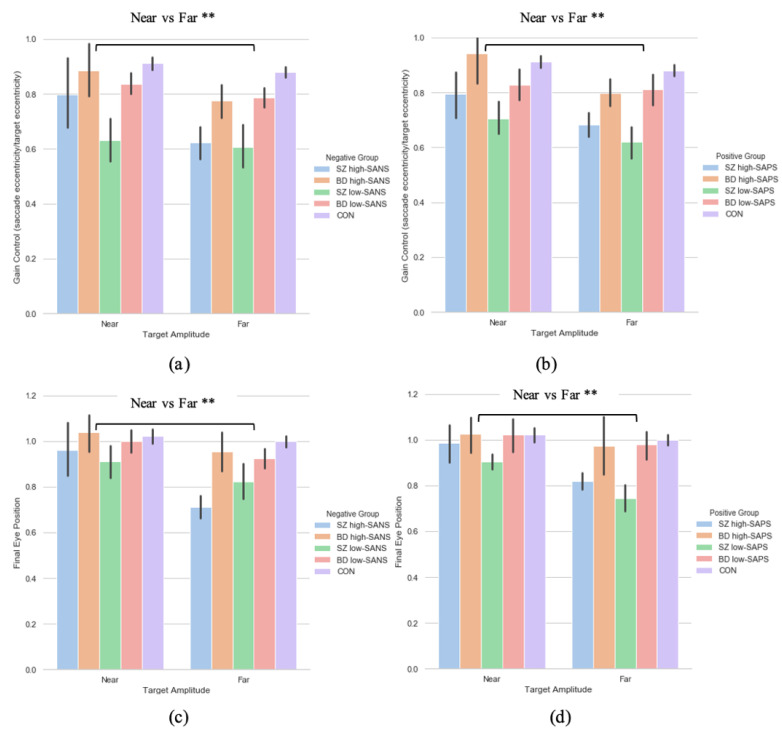
A comparison of performance in gain control for both the (**a**) negative symptom groups and the (**b**) positive symptom groups, as well as performance in the final eye position measure in both the (**c**) negative symptom groups and the (**d**) positive symptom groups, between the two extremities within each diagnostic group. For reference the control cohort are included here but were not included in the reported analysis. ** Refers to significance at the *p* < 0.01 level. Error bars refer to standard error. Error bars refer to standard error.

A two (target eccentricity: Far, Near) by four (group: SZ high-SANS, SZ low-SANS, BD high-SANS, BD low-SANS) repeated measures ANOVA was performed. A significant effect of target eccentricity was found in the gain metric (F(1,59) = 7.51, *p* = 0.008, η_p_^2^ = 0.11; Figure 4a); suggesting reduced gain control for the furthest targets. To explore this potential relationship a multiple regression was performed to predict group membership (high vs. low negative groups, divided by diagnostic label) from gain control towards the furthest target and gain control towards the nearest target. These variables statistically significantly predicted group membership (F(2,60) = 4.65, *p* < 0.01, *R*^2^ = 0.134). Only gain control towards the furthest target added statistically significantly to the prediction, *p* = 0.006. The equivalent repeated-measures ANOVA was carried out for the SAPS groups; illustrating a significant effect of target eccentricity (F(1,60) = 8.29, *p* = 0.006, η_p_^2^ = 0.12; Figure 4b). No group differences were observed for either analysis. A multiple regression was performed to predict group membership (high vs. low positive groups, divided by diagnostic label) from gain control towards the furthest target and gain control towards the nearest target. These variables statistically significantly predicted group membership (F(2,61) = 3.09, *p* = 0.05, *R*^2^ = 0.092). Only gain control towards the furthest target added statistically significantly to the prediction, *p* = 0.02.

#### 3.3.2. Saccade Final Eye Position

A two (target eccentricity: Far, Near) by two (group: high-SANS, low-SANS) repeated a two (target eccentricity: Far, Near) by four (group: SZ high-SANS, SZ low-SANS, BD high-SANS, BD low-SANS) repeated measures ANOVA was carried out. A significant effect of target eccentricity was found in the FEP (F(1,59) = 10.72, *p* = 0.002, η_p_^2^ = 0.15) metric (Figure 4c); suggesting greater undershooting for the furthest targets. To explore this target eccentricity relationship further, a multiple regression was performed to predict group membership (high vs. low negative groups, divided by diagnostic label) from FEP towards the furthest target and FEP towards the nearest target. These variables statistically significantly predicted group membership (F(2,60) = 4.06, *p* = 0.02, *R*^2^ = 0.119). Only FEP towards the furthest target added statistically significantly to the prediction, *p* = 0.007. The equivalent repeated-measures ANOVA analysis was executed for the SAPS groups; illustrating a significant effect of target eccentricity (F(1,60) = 7.89, *p* = 0.007, η_p_^2^ = 0.116; Figure 4d). No group differences were observed for either analysis. A multiple regression was performed to predict group membership (high vs. low positive groups, divided by diagnostic label) from FEP towards the furthest target and FEP towards the nearest target. These variables did not statistically significantly predict group membership (F(2,61) = 2.86, *p* = 0.06, *R*^2^ = 0.086). Only FEP towards the furthest target added statistically significantly to the prediction, *p* = 0.026.

#### 3.3.3. Saccade Latency

A two (target eccentricity: Far, Near) by four (group: SZ high-SANS, SZ low-SANS, BD high-SANS, BD low-SANS) repeated measures ANOVA was carried out; showing no significant effect of the latency metric. The equivalent analysis was carried out for the SAPS groups, illustrating no main effects.

### 3.4. Neuroleptic Medication on Symptomalogy

**Figure 5:**
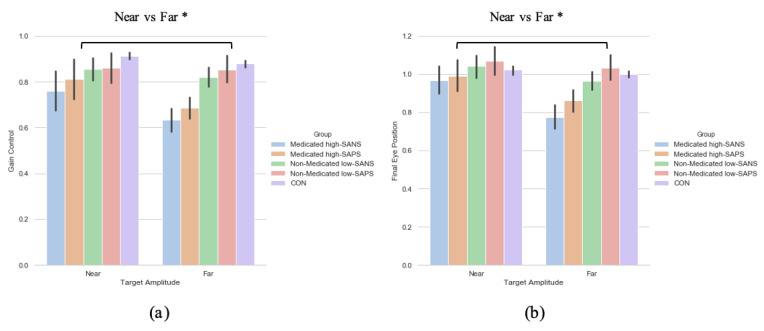
A comparison of performance in the (**a**) gain and (**b**) final eye position measures between the two target eccentricities and the high-/low- SANS/SAPS groups (regardless of diagnostic category). For reference, the control cohort are included here but were not included in the reported analysis. * refers to significance at the *p* < 0.05 level. Error bars refer to the standard error.

To explore the effect of neuroleptic medication on symptomatology—when collapsed across diagnostic category—a two (target eccentricity: Far, Near) by four (group: medicated high-SANS, medicated low-SANS, non-medicated high-SANS, non-medicated low-SANS) repeated measures ANOVA was executed and highlighted no significant differences in the latency metric, but a main effect of target eccentricity in both the gain (F(1,59) = 4.686, *p* = 0.034, η_p_^2^ = 0.074) and FEP (F(1,59) = 6.201, *p* = 0.016, η_p_^2^ = 0.095) measures (see Figure 5a). A two (target eccentricity: Far, Near) by four (group: medicated high-SAPS, medicated low-SAPS, non-medicated high-SAPS, non-medicated low-SAPS) repeated measures ANOVA highlighted no significant differences in the latency metric, but a main effect of target eccentricity in both the gain (F(1,60) = 6.700, *p* = 0.012, η_p_^2^ = 0.100) and FEP (F(1,60) = 9.341, *p* = 0.003, η_p_^2^ = 0.135) measures (see Figure 5a).

In sum, no symptom-based group or target eccentricity differences were observed in relation to the latency metric. This aligns with the data reported in the diagnosis-based analysis. Across analyses exploring the gain control and final eye position metric no group differences were observed. This highlights a divergence from prior literature reporting greater deficit among individuals with more negative symptoms in the memory-guided saccade task [52]. Winograd-Gurvich et al. [52] reported these effects among percentage of errors; thus, gain control illustrates a distinctly different story. Throughout the reported symptom-based analyses, however, greater deficit is observed toward the target with the greatest eccentricity. The analysis investigating the influence of neuroleptic medication illustrated a clear distinction in gain control and final eye position data between target eccentricities: with greater hypometric performance observed toward the furthest eccentricity.

### 3.5. Symptomalogy on Error Rates

**Figure 6:**
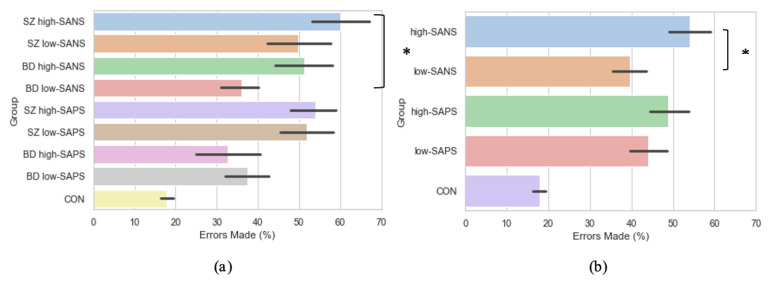
A comparison of error production across the memory-guided saccade paradigm in (**a**) diagnostic groups divided by symptomatology, and (**b**) symptom groups (regardless of diagnostic criteria). For reference, the control cohort are included here but were not included in the reported analysis. * Refers to significance at the *p* < 0.05 level. Error bars refer to the standard error.

Crawford et al. [23,44] reported significantly more errors were exhibited by the schizophrenia patients when compared to bipolar affective disorder patients and control participants. Furthermore, Winograd-Gurvich et al. [52] described how a high-negative symptom group made a significantly greater percentage of errors than controls. Thus, to explore the influence of symptomatology on error rate production, groups were collapsed into high-SANS (*n* = 36) and low-SANS (*n* = 38) categories, regardless of diagnostic category. This was also true for high-SAPS (*n* = 37) and low-SAPS (*n* = 38). When investigated in this manner, the high-SANS group were shown to produce significantly more errors than the low-SANS (F(1,72) = 4.93, *p* = 0.03), but this was not true of the high-/low- SAPS groups (F(1,73) = 0.55, *p* = 0.46; Figure 6b). See the Supplementary Table S2 for all descriptive statistics. This provides support for previous literature [52,53], fostering further the notion that psychotic saccadic deficits may be driven by those individuals experiencing predominantly negative symptoms.

As per the symptom-based analyses reported beforehand, the diagnostic groups were also divided using a median-split into those with high and low negative/positive symptoms: SZ high-SANS (*n* = 17), SZ low-SANS (*n* = 12), BD high-SANS (*n* = 14), BD low-SANS (*n* = 31), SZ high-SAPS (*n* = 26), SZ low-SAPS (*n* = 19), BD high-SAPS (*n* = 8), BD low-SAPS (*n* = 22). Under these conditions, error production was found to differ significantly between the groups (F(3,70) = 3.13, *p* = 0.03; Figure 6a), whereby significantly more errors were produced by the SZ high–SANS cohort compared to the BD low-SANS cohort (*p* = 0.03). No significant differences were found for the same analysis with high- and low- SAPS (F(3,71) = 2.27, *p* = 0.09).

## 4. Discussion

To our knowledge, this is the first study of memory-guided saccades to explore target eccentricity in relation to psychosis diagnosis, psychotic symptomatology and the effect of medication. In a recent paper, greater errors were produced by SZ patients [43] in response to further targets (12°), when compared to near target (6°), although no exploration was given toward the breakdown of symptomatology in this patient group. The present research, therefore, expanded this finding to explore the effect of target eccentricity across diagnostic groups, and between symptom-types. The importance of eccentricity in saccade paradigms relates to the mammalian cortical magnification factor. The representation of retinal ganglion cells is disproportionately increased for foveal compared to peripheral retinal cells and proportional to the distance from the foveal region. Thus, the projections and resolution from 15° will be substantially reduced compared to 7.5°. Therefore, 15° activation is likely to be generate a *weaker* cortical representation, and therefore, potentially more vulnerability to the psychopathology of psychosis. Moreover, larger saccades require greater energy expenditure, with larger neural pulse-steps to generate increased peak velocity and saccade duration for larger saccades. This increased energy demand may make larger saccades, and in the context of the present research, memory representation, more vulnerable to psychopathology.

The current analyses focused on two questions, (1) how do psychotic disorders (schizophrenia and bipolar affective disorder) respond when presented with targets of differing eccentricity in the memory-guided saccade task, and how does medication influence this performance, and (2) how do patients experiencing predominantly negative or predominantly positive symptoms perform?

Data demonstrated no significant group differences when utilising the latency metric; replicating the existing research carried out by Schwartz et al. [42] and Landgraf et al. [26]. With regards to gain control and final eye position, group differences became apparent, highlighting greater deficit among medicated schizophrenia patients and a generally larger impairment towards the furthest target. This finding contrasts the existing literature, whereby deficits in gain control were reported to be prominent among patients with a family history of schizophrenia at smaller angles [42]. However, Schwartz and colleagues found reduced gain at smaller angles (5°–10°) in those patients with a family history of schizophrenia, whereas, at larger angles (16°–30°) both patients with and without a family history of schizophrenia exhibited reduced gain control compared to controls. Thus, the present research is consistent with the relationship shown by Schwartz et al. [42] at the far targets. This is also supported by a recent study by Norouzi et al. [43] who highlighted, among a neurotypical sample, that greater quantities of errors are made toward a target with a greater eccentricity (12°).

Similarly, in relation to symptomatology, greater hypometria and reduced gain control was observed toward the furthest targets. Throughout the symptom-based analyses, greater deficit is observed toward the target with the greatest eccentricity; although, although no group differences were reported throughout the symptom-based analyses. To explore the idea that the far target may be the better predictor of deficit among these participant groups, multiple regressions showed that when exploring symptomatology (divided by diagnostic category, i.e., SZ high-SANS vs. BD high SANS), only the furthest targets significantly accounted for unique group variance in both the eccentricity gain and FEP measures. This, however, was only true when diagnostic category was used to divide the symptom-based groups. When high-/low- SANS groups were collapsed across diagnoses, these significant predictions withdrew. The analysis investigating the influence of medication illustrated a clear distinction in gain control and final eye position data between target eccentricities: with greater hypometric performance observed toward the furthest eccentricity and greater deficit for those medicated patients.

In addition to the saccade metrics reported, the present research also explored error production between groups and symptomatology. A series of studies [52,53,54] reported that greater saccade deficit was observed among individuals who presented with predominantly negative symptoms. Specifically, a greater proportion of errors have been reported among those with high-negative symptoms [52]; prompting the investigation of error production among the current patient cohort. When symptomatology was explored by collapsing all diagnostic categories into predominantly high/low negative/positive symptoms, the high-SANS group were shown to produce significantly more errors than the low-SANS, supporting existing hypotheses. Further, when symptomatology was explored considering diagnostic criteria as well, significantly more errors were produced by the SZ high-SANS cohort and the BD high-SANS cohort in comparison to their corresponding low-SANS cohorts. No such relationship was found for the comparative positive symptoms. This strengthens the notion that saccade deficits, on this occasion, error production, are driven by negative symptoms.

### Strengths and Limitations

The present research aimed to provide an update on the existing understanding of the memory-guided saccade paradigm, taking into account the methodological limitations in the previous literature. To extend our understanding of memory-guided saccade performance among psychosis patients, we included three measures of oculomotor abilities—latency, gain control, and final eye position—to provide a more comprehensive insight into the relationship of psychotic symptoms and target eccentricity. The present data supports the view that gain control and final eye position are promising predictors of psychosis severity; a notion supported by the lack of main effects observed for the latency measure throughout analyses. Moreover, the present data also argues that when investigating degree of deficit among psychosis-spectrum diagnoses, the inclusion of distance target eccentricities (at least 15°) is important, and that reliance on near target eccentricities will risk a loss in sensitivity. Indeed, when resources are limited, it will be a more efficient approach to include only the furthest targets as they provide a sensitive predictor of clinical impairment.

All of the schizophrenia patients who partook in the research reported by Schwartz et al. [42] and Landgraf et al. [26] were medicated; thus, we sought to explore whether differences in memory-guided saccade performance would occur as a consequence of neuroleptic medication status. The data revealed that the medicated groups (with reference to both diagnostic classification and symptomatology-based analyses) performed with heightened deficit in both gain control and final eye position metrics. This, as far as we are aware, is the only research to explore the influence of medication on memory-guided saccade performance in relation to target eccentricity. Moreover, to extend the investigation reported by Winograd-Gurvich et al. [52], the current research also explored the effect of target eccentricity on symptomatology. Winograd-Gurvich et al. [52] reported (with reference to target laterality) that among a cohort of medicated schizophrenia patients, divided by their experience of negative symptoms (high vs. low), no group differences were reported for gain control or final eye position when comparing the high vs. low symptom groups with controls. This finding was replicated in the present dataset with reference to target eccentricity: no group difference with respect to symptomatology were reported. This suggests that within the memory-guided saccade paradigm, diagnostic classification is more prominent in distinguishing differences in saccade performance than symptomatology.

As well as strengths to the present research, there are also limitations worth noting so they can be addressed when moving forward into future memory-guided saccade explorations. Landgraf et al. [26] reported a reduction in error production between small eccentricities and larger eccentricities, which was not present among patients; the present research displayed analyses exploring overall error production amongst symptom-groups, but dividing the errors between target eccentricities was an analysis we were not able to explore in relation to symptomatology. As the present research relied on secondary data analyses it was not possible to explore errors produced toward near and far targets, but this should be considered a priori in future work. Another constraint on the present research were the differential sized cohorts; the absence of a sample size calculation for the different groups used throughout the series of hypothesis-driven analyses, makes the evaluation of statistical power adequacy difficult. This was highlighted particularly during the symptom-based analyses, where we explored symptomatology—predominantly high-/low-SANS and high-/low-SAPS—within the diagnostic classifications. To combat the cohort imbalance, we reported the above analyses and also collapsed high SANS and low SANS groups across diagnosis to explore the singular influence of positive and negative symptomatology (regardless of diagnostic classification).

## 5. Conclusions

The present research extends our understanding of deficit displayed in the memory-guided saccade task and the influence target eccentricity has on psychotic performance. This study reveals that a greater target eccentricity discriminates better between psychiatric groups, particularly for gain control and final eye position metrics. Extensive research in the last 20 years has substantially advanced our understanding of the essential features for the development of oculomotor biomarkers required for the early diagnosis of psychosis. The current findings build on this work by revealing the importance detailed parameter measurement to optimize the sensitivity of these paradigms.

## Figures and Tables

**Table 1:** Clinical, psychiatric, and neuropsychological profile of medicated (M) and non-medicated (NM) schizophrenic and bipolar patients, and control participants, as well as the current dosage of neuroleptic medication, expressed in chlorpromazine equivalent units (group means and standard deviation).

	Schizophrenia Patients		Bipolar Disorder Patients		Control Participants
	Medicated	Non-Medicated	Medicated	Non-Medicated	
Chlorprozamine equivalent units	1637 (572)	-	1186 (474)	-	-
Age (year)	39.9 (12.3)	39.4 (13.2)	43.6 (12.1)	42.1 (12.3)	38.5 (10.8)
Disease duration (year)	13.7 (10.7)	12.9 (14.8)	20.5 (11.6)	14.7 (13.6)	-
Age of onset (year)	26.3 (8.9)	25.4 (10.6)	23.1 (8.9)	27.4 (12.2)	-
Negative symptoms (SANS)	27.5 (17.1)	21.5 (19.7)	13.7 (17.6)	4.6 (6.8)	1.0 (1.9)
Positive symptoms (SAPS)	16.6 (21.7)	17.7 (17.8)	5.1 (9.2)	3.4 (6.6)	0.3 (0.2)

## Data Availability

The data will be shared publicly via the Lancaster University PURE system.

## Supplementary Materials

The following are available online at https://www.mdpi.com/article/10.3390/brainsci11081071/s1 and https://osf.io/ucgv3/ [55], S1: Details of Clinical Assessments, Apparatus used and Testing Procedures, Table S2: Error Produc-tion Across the Different Cohorts, Investigated based on Diagnostic Criteria and Symptomatology.
