# Supplementary material for: Memory-Guided Saccades in Psychosis: Effects of Medication and Stimulus Location
Source: Brain Sci. 2021 Aug 16;11(8):1071. doi: 10.3390/brainsci11081071 (PMC8393375; doi:10.3390/brainsci11081071)
Supplement: Supplementary file 1 [file brainsci-11-01071-s001.zip › brainsci-1305950-supplementary.pdf]

S1. Details of Clinical Assessments, Apparatus used and Testing Procedures.

Table S1. Details of the particular neuroleptic drugs taken, as well as the other drug treatments by medicated SZ and BD participants.

	M-SZ	M-BD
Haloperidol	4	4
Flupenthixol deconate	15	1
Trifluoperazinc	5	1
Chlorpromazine	3	5
Fluphcnazine deconate	14	3
Sulpuride	1	0
Clopenthixol deconate	2	2
Others	2	1
Procyclidine/orphenadrine	21	8
Lithium	0	7

Clinical Assessment.

The patients were assessed clinically using a structured interview, which included the Schedule for Affective Disorders and Schizophrenia, (SADS; Spitzer & Endicott, 1975), the Scale for the Assessment of Negative Symptoms (SANS; Andreasen, 1984a), the Scale for the Assessment of Positive Symptoms (SAPS; Andreasen, 19846), and the Mini-Mental State Examination (MM; Folstein et al. 1975). Additionally, their cognitive performance was assessed by the National Adult Reading Test (NART; Nelson, 1982), Raven's Progressive Matrices Test (Raven & Court, 1965). The revised Wisconsin Card Sort test (Heaton, 1981) was used to derive a total error score (WCST); the perseveration score (WCSP) was used as our primary measure of frontal dysfunction and inhibitory control. The presence of tardive dyskinesia (TD) was also reported in order to explore the influence of dopamine in predictive saccade responding – of the M-SZ cohort, 35% reported TD (*n*=14), and of the M-BD cohort, 14.3% reported TD (*n*=2). A full neurological examination was also carried out on each patient. See group means and standard deviations on all measures in Table 1 of the article.

Apparatus.

The target display consisted of 4 red LED targets (diameter 0.25°) located ±7.5 and +15° either side of the central fixation LED. The LED targets were embedded in a semi-opaque screen and were only visible when illuminated. Subjects were comfortably seated 1-5 m from the screen with a buzzer located centrally behind the subject's head. Movements of the head were constrained by use of an adjustable headrest. The experiment was conducted in the dark. Eye movements were recorded using

an infra-red limbus-reflection device (Skalar (IRIS) with a linearity range of ±15°. A hardware antialiasing filter (cut-off frequency 200 Hz) was used to filter eye position and the sampling rate was 500 Hz. Blinks were monitored using electro-oculography with electrodes placed above and below one eye. The stimulus display and data sampling were controlled by a PDP 11/73 computer. Saccadic analysis was conducted off-line using interactive software, which enabled the rejection of artefacts due, for example, to blinks. Saccadic detection was based on a velocity criterion of 30°/s in addition to an acceleration across three consecutive samples. Final eye position was measured by taking the mean fixation location during the maximal period of fixation stability after all secondary corrective saccades were completed.

Procedure.

Each subject participated in four saccade paradigms during the experiment, but the present manuscript will focus only on the memory-guided paradigm. The memory-guided saccade block of the paradigm consisted of 48 trials (see Figure 1.).

Memory-Guided Saccade paradigm.

A central LED was illuminated. After 800 milliseconds (ms), a peripheral LED flashed on for 200ms. The central LED remained on; however, the participant was instructed not to look toward the peripheral LED immediately. The central LED was extinguished 500ms after offset of the peripheral target and, at this point, the participant was required to make a saccade to the remembered location of the previously illuminated peripheral target LED. In this paradigm, the buzzer onset that provided the temporal cue, was coincident with the offset of the central (fixation) LED.

Table S2. Error Production across the different cohorts, investigated based on diagnostic criteria and symptomatology.

	n	Mean Error	Standard deviation
SZ high-SANS	17	60.12	29.71
SZ low-SANS	12	49.83	28.54
BD high-SANS	8	61.13	28.67
BD low-SANS	7	36.57	28.50
SZ high-SAPS	26	54.00	29.67
SZ low-SAPS	19	51.82	29.71
BD high-SAPS	8	32.75	23.90
BD low-SAPS	22	37.50	25.18
High-SANS	36	54.06	29.48
Low-SANS	38	39.63	26.38
High-SAPS	37	48.84	29.02
Low-SAPS	38	43.92	28.35
Medicated high-SANS	27	56.59	28.84
Medicated low-SANS	14	49.79	24.81
Non- Medicated high-SANS	9	46.44	31.82
Non- Medicated low-SANS	24	33.71	25.92
Medicated high-SAPS	22	51.23	26.68
Medicated low-SAPS	21	54.19	29.39
Non- Medicated high-SAPS	15	45.33	32.80
Non- Medicated low-SAPS	17	31.24	21.66
